# The relationship between vitamin D levels and Alzheimer’s disease risk: insights from a centenarian study of Chinese women

**DOI:** 10.3389/fnut.2025.1628732

**Published:** 2025-06-20

**Authors:** Yupeng Li, Xujie Wang, Mei Yu, Fei Wang, Dong Song, Musong Liu, Xu Liang, Hongzhou Liu, Jiangbo Liu, Shihui Fu, Xuhui Liu

**Affiliations:** ^1^Diabetes Center and Department of Health Management, NHC Key Lab of Hormones and Development and Tianjin Key Lab of Metabolic Diseases, Tianjin Medical University Chu Hsien-I Memorial Hospital & Institute of Endocrinology, Tianjin, China; ^2^Department of Emergency ICU, The Affiliated Hospital of Qinghai University, Xining, China; ^3^Laboratory Department, Hainan Hospital of Chinese People's Liberation Army General Hospital, Sanya, China; ^4^Department of Orthopedics, Tianjin Haihe Hospital, Tianjin, China; ^5^Department of Endocrinology, Aerospace Center Hospital, Beijing, China; ^6^Department of Clinical Medicine, School of Basic Medical Sciences, Hebei Medical University, Shijiazhuang, China; ^7^Tianjin Eye Hospital, Tianjin Key Lab of Ophthalmology and Visual Science, Tianjin Eye Institute, Tianjin, China; ^8^Respiratory Department, Tianjin First Central Hospital, Tianjin, China; ^9^Department of Cardiology, Hainan Hospital of Chinese People's Liberation Army General Hospital, Hainan Geriatric Disease Clinical Medical Research Center, Hainan Branch of China Geriatric Disease Clinical Research Center, Sanya, China; ^10^Department of Geriatric Cardiology, Chinese People's Liberation Army General Hospital, Beijing, China; ^11^Neurology Department, The Second Hospital of Lanzhou University, Lanzhou, China

**Keywords:** vitamin D3, Alzheimer’s disease, centenarians, cognitive aging, neurodegenerative risk

## Abstract

**Background:**

While vitamin D₃ (VD₃) has been implicated in Alzheimer’s disease (AD) prevention, limited evidence exists among centenarians—particularly women—who exhibit unique cognitive aging trajectories. This study aimed to examine the association between serum 25-hydroxyvitamin D [25(OH)D] levels and AD risk in Chinese female centenarians.

**Methods:**

We included 514 female participants aged ≥100 years from the China Healthy Longevity Multicenter Study (CHLMS). AD was diagnosed using education-adjusted MMSE thresholds and clinical exclusion of non-AD dementias. Serum 25(OH)D and biochemical markers were measured using standardized laboratory protocols. Logistic regression models (unadjusted and progressively adjusted) assessed associations between 25(OH)D and AD. Restricted cubic spline (RCS) and piecewise regressions evaluated non-linear and threshold effects, while subgroup analyses explored effect modification.

**Results:**

Higher serum 25(OH)D levels were independently associated with lower odds of AD (adjusted OR per 1 ng/mL: 0.95; 95% CI: 0.90–1.00; *p* = 0.037). Compared to the lowest quartile, participants in the highest quartile had an 87% reduced risk (OR = 0.13; 95% CI: 0.03–0.50; *p* = 0.007). RCS analysis revealed a significant inverse dose–response relationship, with a potential threshold effect observed at 29.3 ng/mL. Piecewise regression confirmed that the protective association was strongest below this threshold. Subgroup analyses across smoking, hypertension, and early-life indicators showed consistent effects with no significant interactions.

**Conclusion:**

Among Chinese female centenarians, serum vitamin D₃ levels are inversely associated with AD risk in a dose-dependent manner, particularly below 29.3 ng/mL. These findings highlight the relevance of vitamin D₃ as a potentially modifiable factor in cognitive aging and support further interventional studies in the oldest-old population.

## Introduction

Alzheimer’s disease (AD), the leading cause of dementia, has emerged as a global public health crisis, with an estimated 55 million affected individuals worldwide, 60–70% of whom suffer from AD pathology (1). In China, rapid population aging has escalated AD prevalence to over 10 million cases, projected to triple by 2050, imposing a staggering socioeconomic burden equivalent to 1.5% of GDP (2). Beyond its devastating cognitive decline, AD disproportionately strains healthcare systems and informal caregivers, accounting for $1.3 trillion in annual global costs (3). Notably, age remains the strongest non-modifiable risk factor: AD incidence doubles every 5 years after age 65, reaching 40% among individuals aged ≥90 (4). Centenarians—now the fastest-growing age group globally with a 12% annual increase—represent a unique population to study resilience against age-related neurodegeneration (5). However, centenarians—a rapidly growing demographic—exhibit remarkable heterogeneity in AD susceptibility. Emerging evidence suggests that female centenarians, who constitute 85% of the global centenarian population, may harbor distinct genetic and epigenetic adaptations (e.g., FOXO3A variants, attenuated mTOR signaling) that decouple chronological age from AD risk (6). Intriguingly, some centenarians maintain intact cognition despite carrying high-risk APOE ε4 alleles (7), suggesting age-specific resilience mechanisms that could inform novel therapeutic strategies.

Vitamin D (VD) has garnered attention as a neuroprotective agent. Epidemiological evidence further links low serum 25-hydroxyvitamin D [25(OH)D] (<20 ng/mL) to a 2.3-fold increased AD risk (8), with randomized trials showing VD supplementation slows cognitive decline in mild cognitive impairment (MCI) (9). Notably, sex-specific responses to VD supplementation have been reported: postmenopausal women exhibit greater cognitive benefits from VD than men, potentially due to estrogen-VDR crosstalk (10). Despite these advances, critical gaps persist. First, centenarians—particularly women—are excluded from 98% of VD-AD studies (11), creating a critical evidence gap given their distinct VD metabolism (e.g., reduced dermal synthesis capacity, chronic low-grade inflammation) (12). Second, residual confounding from unmeasured variables—e.g., educational attainment, early-life nutritional deprivation, or epigenetic modifications—undermines causal inference (13). For female centenarians, early-life adversities (e.g., gender-based nutritional disparities) may compound VD deficiency across the lifespan (14). For instance, adjusting for education attenuates VD-AD associations by 30% (15), while famine-exposed cohorts exhibit lifelong VD deficiency and elevated AD risk (16). Third, optimal VD thresholds remain controversial.

To address the dual knowledge gaps in centenarian and female AD research, we leverage the China Healthy Longevity Multicenter Study (CHLMS), the world’s largest prospective cohort of centenarians and oldest-old individuals, to investigate 25(OH)D-AD associations in extreme longevity. Focusing on female centenarians addresses a critical public health priority: women bear 65% of the global AD burden, yet sex-stratified analyses in VD trials remain rare (17). Our female-centric design also captures menopause-related VD dynamics: 95% of women experienced natural menopause before age 50, offering insights into prolonged postmenopausal VD exposure (18). Our findings challenge the dogma of “age-dependent VD efficacy loss” and could redefine VD supplementation guidelines for the oldest-old, advancing precision prevention in aging populations.

## Methods

### Study population and data collection

The study population was derived from the CHLMS, a large-scale population-based cohort. This cohort involved a comprehensive household survey targeting all centenarians and oldest-old individuals, identified via official registries provided by the Ministry of Civil Affairs of China. For the present analysis, participants were excluded if their Mini-Mental State Examination (MMSE) data were missing or if serum vitamin D₃ levels were not measured. We further restricted the sample to female participants aged ≥100 years. Ultimately, the final study population consisted of 514 female centenarians who underwent standardized home interviews, physical examinations, and blood sample collection and analysis. Data collection was conducted by a systematically trained multidisciplinary team composed of geriatricians, endocrinologists, and nursing staff. All interviews were conducted in person using unified operational protocols, adhering strictly to international standards for clinical research quality control. Demographic information such as age and sex was verified using second-generation resident identification card scanners to eliminate manual entry errors (accuracy: 99.98%). All data were stored in encrypted formats on databases compliant with the Health Insurance Portability and Accountability Act (HIPAA, 2023 Edition) to ensure data privacy and security. The study protocol was approved by the Ethics Committee of Hainan Hospital, General Hospital of the People’s Liberation Army of China (Approval No. 301HN11201601), and was conducted in accordance with the Declaration of Helsinki. Written informed consent was obtained from all participants or their legally authorized representatives.

### Outcome definition

The primary outcome of this study was the presence of Alzheimer’s disease (AD), defined based on a composite framework incorporating clinical symptoms, cognitive performance, and the exclusion of alternative diagnoses. Diagnosis adhered to criteria adapted from both international guidelines (e.g., NIA-AA, DSM-IV) and large-scale Chinese epidemiological studies. Specifically, participants were diagnosed with AD if they (1) presented with characteristic symptoms such as progressive memory impairment, language decline, and executive dysfunction; and (2) exhibited cognitive impairment according to the Mini-Mental State Examination (MMSE), with education-adjusted cutoffs: ≤17 for illiterate individuals, ≤20 for primary school education, ≤22 for secondary or technical school education, and ≤23 for college or higher education. Participants were excluded from the AD group if they met criteria for other dementia types—such as vascular dementia, Parkinson’s disease dementia, or frontotemporal dementia—based on their medical history, clinical examination, and neurologist adjudication. Additional exclusion criteria included: (1) acute delirium or psychiatric conditions that could mimic dementia; (2) major stroke within 6 months prior to assessment; and (3) missing or invalid MMSE scores. Individuals meeting the above AD criteria were classified as AD-positive, while all others were considered AD-negative for subsequent analysis.

### Laboratory measurements

Fasting venous blood samples were collected in the morning by experienced nurses using disposable vacuum-sealed tubes under standardized venipuncture protocols. All samples were transported to the central laboratory within 4 h and stored at 4°C prior to analysis. Serum 25-hydroxyvitamin D [25(OH)D] concentrations were measured using enzymatic assays on the Cobas analyzer (Roche Diagnostics, Basel, Switzerland). The same platform was employed for the assessment of osteocalcin (OST) and Cross-linked C-telopeptide of Type I Collagen (CTX), as well as intact parathyroid hormone (PTH), ensuring consistency in assay sensitivity and standardization. Additional biochemical parameters—including creatinine (μmol/L), calcium (mmol/L), phosphorus (mmol/L), magnesium (mmol/L), glucose (mmol/L), and serum iron (μg/dL)—were determined using enzymatic methods validated for clinical use. Potassium (mmol/L) and Fructosamine (µmol/L) were assayed enzymatically. Immunoglobulin kappa-associated protein (IGKAP, pg./mL) and procollagen type I N-terminal propeptide (PINP, μg/L) were quantified using enzymatic assays. All laboratory tests were conducted under rigorous internal and external quality control procedures to ensure analytical accuracy and reproducibility.

### Clinical history and scale definitions

Clinical history and structured scale-based variables were collected through standardized interviews to capture participants’ long-term exposures and background characteristics. Smoking status was categorized as “Yes” or “No” based on self-reported current or former tobacco use. Hypertension was defined as SBP ≥ 140 mmHg, DBP ≥ 90 mmHg or current use of antihypertensive medications. To assess life-course socioeconomic and physical status, six retrospective self-reported variables were included in the analysis. Childhood body type (B8A) was determined by asking participants to evaluate their body size before age 14 compared to peers, and responses were dichotomized as 1 = overweight and 0 = not overweight. Childhood socioeconomic status (B8B) was assessed based on participants’ perception of their family’s economic situation before age 14, categorized as 1 = poor and 0 = good. Similarly, adolescent body type (B9A), referring to ages 14–30, was coded as 1 = overweight and 0 = not overweight based on self-comparison with age-matched peers, while adolescent socioeconomic status (B9B) was classified as 1 = poor and 0 = good. Adult body type (B10A), reflecting perceived body size between ages 30 and 60, was recorded as 1 = overweight and 0 = not overweight, and adult socioeconomic status (B10B) during the same age span was coded as 1 = poor and 0 = good. All variables were dichotomized for analytical consistency, with higher scores reflecting adverse physical or socioeconomic conditions. These life-course indicators, adapted from validated epidemiological instruments, were used in subsequent analyses to account for potential early-life determinants of cognitive and metabolic outcomes.

### Statistical analyses

Baseline characteristics were summarized using means ± standard deviations for continuous variables and frequencies (percentages) for categorical variables. Group comparisons between participants with and without Alzheimer’s disease (AD) were assessed using Student’s t-tests and chi-square tests, respectively. To evaluate the association between serum 25-hydroxyvitamin D [25(OH)D] levels and AD, multivariable logistic regression models were constructed with progressive adjustments: Model 1 was unadjusted; Model 2 adjusted for age, smoking status, hypertension, and early-life indicators (B8A–B10B); and Model 3 further adjusted for biochemical markers of renal function, mineral and glucose metabolism, bone turnover, and inflammation. Vitamin D levels were analyzed both continuously (per 1 ng/mL increase) and as quartiles, with the lowest quartile as reference. Linear trend across quartiles was tested using median values as a continuous variable. Restricted cubic spline (RCS) regression with three knots was used to explore non-linear associations, with the median 25(OH)D level as the reference. Threshold effects were assessed using piecewise logistic regression with a breakpoint at 29.3 ng/mL, and model fit was compared using a likelihood ratio test. Sensitivity analyses were performed via subgroup logistic regressions stratified by smoking, hypertension, and B8A–B10B categories, and potential interactions were tested; *p*-values for interaction >0.05 were interpreted as no significant effect modification. All analyses were conducted using R version 4.4.2, with a two-sided *p* < 0.05 considered statistically significant.

## Results

Table 1 summarizes the baseline characteristics of the study cohort stratified by Alzheimer’s disease (AD) status (*n* = 514; AD-positive: 467, AD-negative: 47). Compared to the AD-negative group, individuals with AD exhibited significantly lower serum levels of vitamin D₃ (21 ± 7 vs. 24 ± 6 ng/mL, *p* < 0.001), calcium (2.21 ± 0.11 vs. 2.25 ± 0.12 mmol/L, *p* = 0.014), iron (11.5 ± 4.2 vs. 13.1 ± 5.2 μg/dL, *p* = 0.040), and Potassium (mmol/L) (4.49 ± 0.70 vs. 4.77 ± 0.77 U/L, *p* = 0.019), but higher levels of IGKAP (431 ± 120 vs. 399 ± 94 pg./mL, *p* = 0.036) and procollagen type I N-terminal propeptide (PINP, 80 ± 43 vs. 68 ± 31 μg/L, *p* = 0.026). Additionally, the AD-positive group was slightly older (102.96 ± 2.94 vs. 102.15 ± 2.26 years, *p* = 0.027). Categorical variables revealed significantly higher positivity rates of B8B (*p* < 0.001) and B9B (*p* = 0.001) in the AD group. No significant differences were found for creatinine, phosphorus, glucose, magnesium, Fructosamine (µmol/L), osteocalcin (OST), cross-linked C-telopeptide of type I collagen (CTX), or parathyroid hormone (PTH), nor in smoking status, hypertension, or the expression of B8A, B9A, B10A, and B10B.

**Table 1:** Baseline characteristics of the study population stratified by Alzheimer’s disease (AD) status.

Characteristic	AD		*p*-value
	0, *N* = 47	1, *N* = 467	
Vitamin D3 (ng/mL)	24 ± 6	21 ± 7	<0.001
Age (years)	102.15 ± 2.26	102.96 ± 2.94	0.027
Creatinine (μmol/L)	78 ± 30	81 ± 31	0.520
Phosphorus (mmol/L)	1.10 ± 0.13	1.08 ± 0.17	0.344
Calcium (mmol/L)	2.25 ± 0.12	2.21 ± 0.11	0.014
Iron (μg/dL)	13.1 ± 5.2	11.5 ± 4.2	0.040
Glucose (mmol/L)	4.90 ± 1.09	5.18 ± 1.53	0.109
Magnesium (mmol/L)	0.88 ± 0.10	0.89 ± 0.11	0.455
Potassium (mmol/L)	4.77 ± 0.77	4.49 ± 0.70	0.019
Fructosamine (µmol/L)	262 ± 25	262 ± 29	0.988
IGKAP (pg/mL)	399 ± 94	431 ± 120	0.036
OST (ng/mL)	33 ± 15	35 ± 23	0.230
PINP (μg/L)	68 ± 31	80 ± 43	0.026
CTX (ng/mL)	0.45 ± 0.30	0.46 ± 0.27	0.805
PTH (pg/mL)	45 ± 23	51 ± 29	0.110
Smoke			0.457
No	44 (93.6%)	447 (95.7%)	
Yes	3 (6.4%)	20 (4.3%)	
Hypertension			0.599
No	34 (72.3%)	354 (75.8%)	
Yes	13 (27.7%)	113 (24.2%)	
B8A			0.425
No	41 (87.2%)	386 (82.7%)	
Yes	6 (12.8%)	81 (17.3%)	
B8B			<0.001
No	11 (23.4%)	234 (50.1%)	
Yes	36 (76.6%)	233 (49.9%)	
B9A			0.182
No	43 (91.5%)	393 (84.2%)	
Yes	4 (8.5%)	74 (15.8%)	
B9B			0.001
No	10 (21.3%)	212 (45.4%)	
Yes	37 (78.7%)	255 (54.6%)	
B10A			>0.999
No	43 (91.5%)	427 (91.4%)	
Yes	4 (8.5%)	40 (8.6%)	
B10B			0.060
No	6 (12.8%)	117 (25.1%)	
Yes	41 (87.2%)	350 (74.9%)	

OST: Osteocalcin; PINP: Procollagen Type I N-Terminal Propeptide; CTX: Cross-linked C-telopeptide of Type I Collagen; PTH: Parathyroid Hormone; B8A: Childhood body type; B8B: Childhood socioeconomic status; B9A: adolescent body type; B9B: adolescent socioeconomic status; B10A: Adult body type; B10B: adult socioeconomic status.

To evaluate the relationship between serum vitamin D₃ (VD₃) levels and the likelihood of Alzheimer’s disease (AD), we developed three logistic regression models with progressive adjustment for potential confounders. Model 1 was unadjusted. Model 2 included demographic and early-life indicators such as smoking, hypertension, age, and categorical variables B8A, B8B, B9A, B9B, B10A, and B10B. Model 3 further adjusted for a comprehensive set of biochemical covariates, including renal function (Cr), mineral metabolism (Ca, P, Iron, Mg), glucose metabolism (Glucose), bone turnover markers (OST, CTX, TINP, PTH), and inflammatory indicators (IGKAP). As shown in Table 2, in Model 1, each 1 ng/mL increase in serum VD₃ was associated with a 6% reduction in AD risk (OR = 0.94, 95% CI: 0.90–0.98, *p* = 0.003). This inverse association remained stable after adjustment in Model 2 (OR = 0.94, 95% CI: 0.90–0.98, *p* = 0.007) and persisted in Model 3 (OR = 0.95, 95% CI: 0.90–1.00, *p* = 0.037). When modeled as quartiles, higher VD₃ levels were consistently associated with reduced AD risk across all models. Compared to the lowest quartile (Q1: 3.9–16 ng/mL), individuals in Q2–Q4 exhibited lower odds of AD, with a clear dose–response trend. In Model 1, the odds ratios for Q2, Q3, and Q4 were 0.23 (95% CI: 0.05–0.75, *p* = 0.026), 0.22 (95% CI: 0.05–0.71, *p* = 0.021), and 0.14 (95% CI: 0.03–0.44, *p* = 0.002), respectively. These protective effects remained significant in Model 2 (Q2: OR = 0.22, *p* = 0.025; Q3: OR = 0.21, *p* = 0.020; Q4: OR = 0.15, *p* = 0.004), and were still observed in the fully adjusted Model 3 (Q2: OR = 0.16, *p* = 0.015; Q3: OR = 0.17, p = 0.021; Q4: OR = 0.13, *p* = 0.007). A significant linear trend was noted in all three models (p-trend = 0.002, 0.004, and 0.017, respectively), supporting a dose-dependent inverse relationship between serum VD₃ and AD risk.

**Table 2:** Association between serum vitamin D₃ levels and risk of Alzheimer’s disease.

Characteristic	Model 1			Model 2			Model 3		
	OR^1^	95% CI^1^	*p*-value	OR^1^	95% CI^1^	*p*-value	OR^1^	95% CI^1^	*p*-value
VD3 (continuous)	0.94	0.90, 0.98	0.003	0.94	0.90, 0.98	0.007	0.95	0.90, 1.00	0.037
VD3									
Q1 [3.9,16]	—	—		—	—		—	—	
Q2 [16,21.2]	0.23	0.05, 0.75	0.026	0.22	0.05, 0.74	0.025	0.16	0.03, 0.61	0.015
Q3 [21.2,26.1]	0.22	0.05, 0.71	0.021	0.21	0.05, 0.70	0.020	0.17	0.03, 0.67	0.021
Q4 [26.1,40.2]	0.14	0.03, 0.44	0.002	0.15	0.04, 0.48	0.004	0.13	0.03, 0.50	0.007
P for trend			0.002			0.004			0.017

1 OR, Odds Ratio; CI. Confidence Interval.

Model 1: no covariates were adjusted; Model 2: adjusted for Smoke, Hypertension, B8A, B8B, B9A, B9B, B10A, B10B, and Age; Model 3: adjusted for Smoke, Hypertension, early-life indicators (B8A, B8B, B9B, B10A, B9A, B10B), Age, Cr, Mg, Ca, P, Iron, Glucose, K, Fructosamine, IGKAP, OST, CTX, PINP, and PTH.

### Dose–response relationship between serum vitamin D₃ and Alzheimer’s disease risk

**Figure 1:**
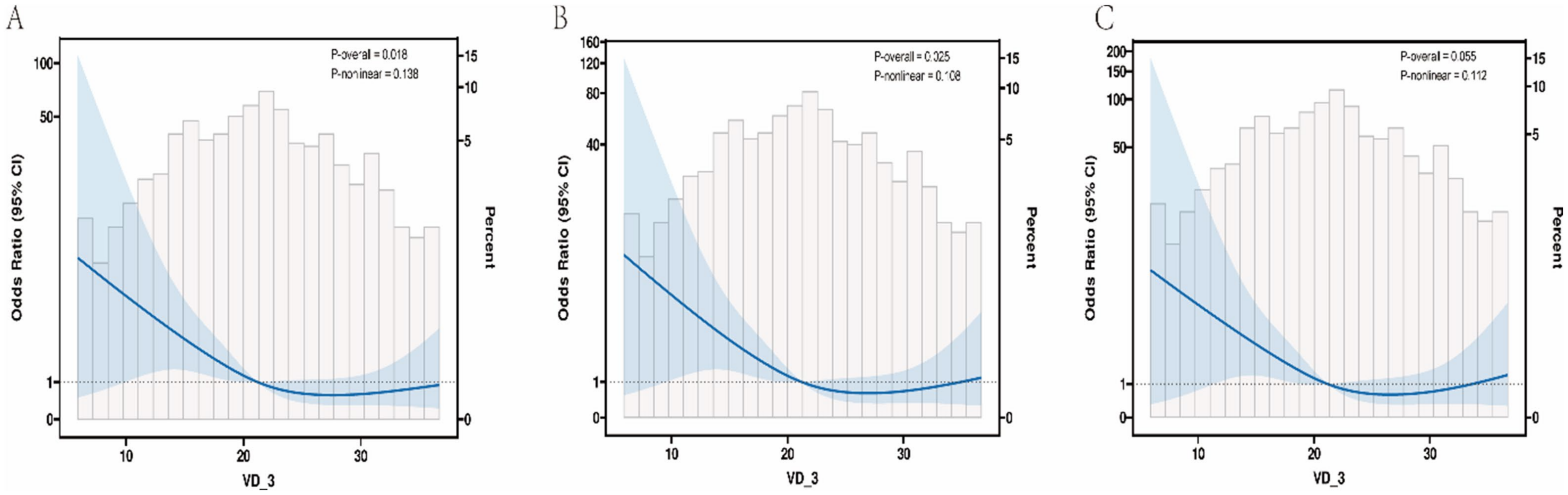
**(A)** Model 1: unadjusted; **(B)** Model 2: adjusted for age, smoking status, hypertension, and early-life indicators (B8A, B8B, B9A, B9B, B10A, B10B); **(C)** Model 3: adjusted for age, Smoke status, Hypertension, early-life indicators(B8A, B8B, B9B, B10A, B9A, B10B), Cr, Mg, Ca, P, Iron, Glucose, K, Fructosamine, IGKAP, OST, CTX, PINP, and PTH.

Restricted cubic spline (RCS) plots depicting the dose–response relationship between serum vitamin D₃ levels and the odds of Alzheimer’s disease (AD) under three logistic regression models. Solid lines represent adjusted odds ratios (ORs), and shaded bands indicate 95% confidence intervals. The reference value (OR = 1.0) is set at the median serum VD₃ level. To further examine the potential non-linear relationship between serum vitamin D₃ (VD₃) levels and AD risk, restricted cubic spline (RCS) regression analyses were performed using the three previously defined logistic models. As shown in Figure 1, all three spline curves consistently demonstrated an inverse association between VD₃ levels and the odds of AD. In Model 1 (unadjusted), a clear monotonic and approximately linear inverse association was observed, with a marked decline in AD risk at lower VD₃ concentrations and a plateau at higher levels. This pattern remained evident in Model 2 after adjustment for age, smoking status, hypertension, and early-life factors (B8A–B10B), although the slope of the decline was modestly attenuated. In the fully adjusted Model 3, which incorporated a comprehensive panel of biochemical covariates, the inverse association persisted, albeit with further attenuation beyond approximately 30 ng/mL, suggesting a potential threshold effect. Importantly, none of the models indicated a U-shaped or J-shaped curve, thereby reinforcing a consistent, monotonic protective effect of increasing VD₃ levels against AD. These RCS-derived findings corroborate the associations observed in both the continuous and categorical regression analyses and further support a dose-dependent inverse relationship, particularly within the low to moderate range of serum VD₃ (<30 ng/mL).

### Threshold effect of serum vitamin D₃ on Alzheimer’s disease risk

To assess whether the association between serum vitamin D₃ (VD₃) levels and Alzheimer’s disease (AD) risk follows a threshold-dependent pattern, a piecewise logistic regression model was applied, using 29.3 ng/mL as the inflection point determined from the data. As shown in Table 3, below the threshold of 29.3 ng/mL, VD₃ was significantly associated with reduced odds of AD (OR = 0.89, 95% CI: 0.83–0.96, *p* = 0.002). In contrast, above the threshold (VD₃ ≥ 29.3 ng/mL), no statistically significant association was observed (OR = 1.15, 95% CI: 0.88–1.50, *p* = 0.303). The likelihood ratio test comparing the piecewise and standard logistic models yielded a borderline *p*-value of 0.065, suggesting a potential but not definitive improvement in model fit when allowing for a threshold effect.

**Table 3:** Threshold effect analysis of serum vitamin D₃ on Alzheimer’s disease risk using piecewise logistic regression.

Model	OR (95% CI)	*p*-value
Standard logistic regression	0.94 (0.90, 0.98)	0.003
Piecewise logistic regression (Breakpoint = 29.3 ng/mL)		
VD₃ < 29.3 ng/mL	0.89 (0.83, 0.96)	0.002
VD₃ ≥ 29.3 ng/mL	1.15 (0.88, 1.50)	0.303
Log-likelihood ratio test	—	0.065

### Subgroup analyses and sensitivity assessment

**Figure 2:**
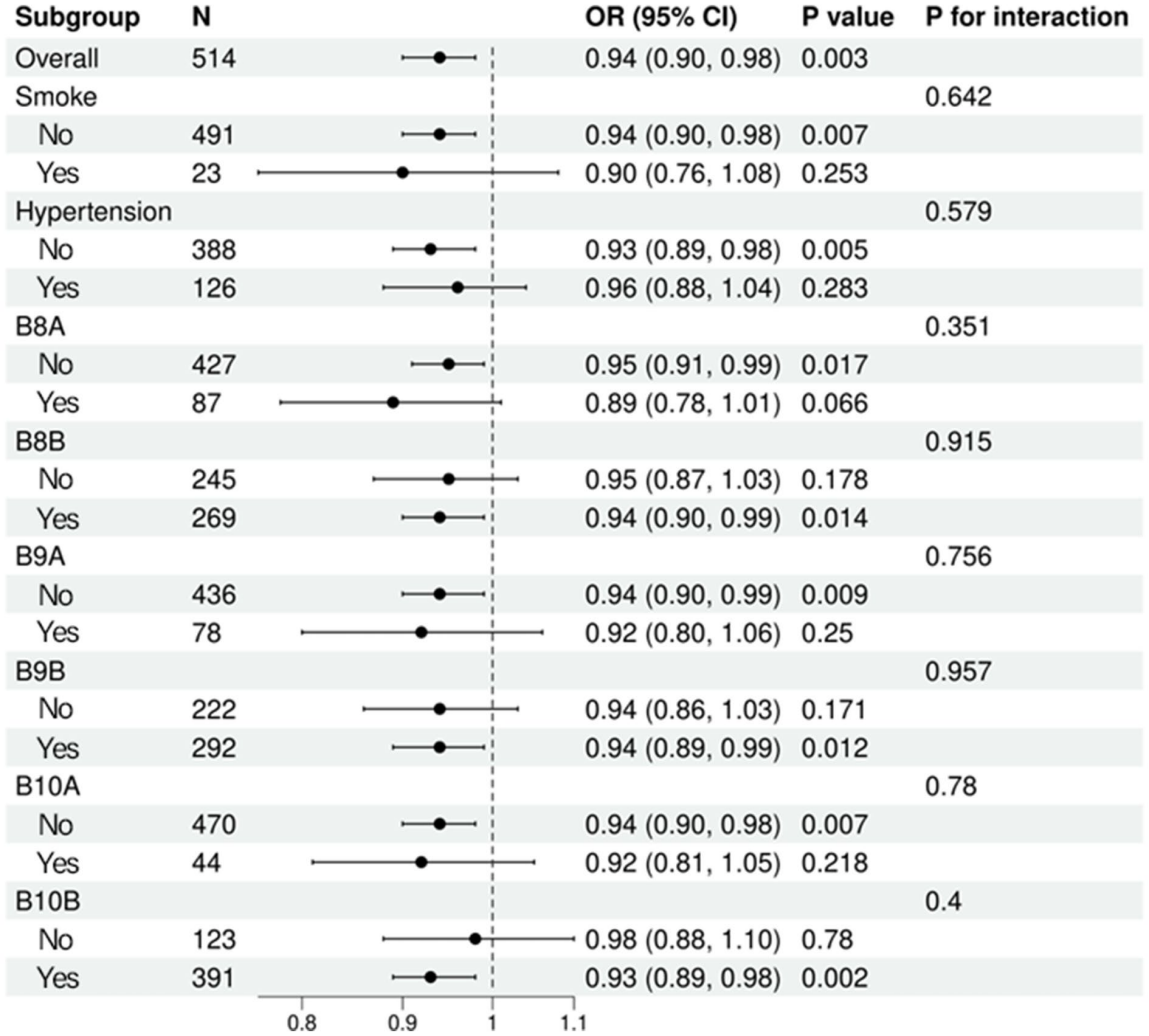
Forest plot of subgroup analyses for the association between serum vitamin D₃ and Alzheimer’s disease.

To examine the robustness and potential effect modification of the association between serum vitamin D₃ (VD₃) levels and Alzheimer’s disease (AD), we conducted stratified logistic regression analyses across key subgroups. These subgroups were defined by smoking status, hypertension history, and six early-life indicators (B8A, B8B, B9A, B9B, B10A, B10B), representing body size and socioeconomic status during childhood. The results are presented in Figure 2. The inverse association between VD₃ and AD risk remained broadly consistent across all subgroups. Statistically significant protective effects of VD₃ were observed in non-smokers (OR = 0.94, 95% CI: 0.90–0.98, *p* = 0.007), participants without hypertension (OR = 0.93, 95% CI: 0.89–0.98, *p* = 0.005), and several early-life subpopulations, including B8A-negative (OR = 0.95, *p* = 0.017), B8B-positive (OR = 0.94, *p* = 0.014), B9A-negative (OR = 0.94, *p* = 0.009), B9B-positive (OR = 0.94, *p* = 0.012), B10A-negative (OR = 0.94, p = 0.007), and B10B-positive individuals (OR = 0.93, *p* = 0.002). Importantly, no significant interaction effects were identified (all p for interaction > 0.05), indicating that the association between VD₃ and AD was homogeneous across all examined strata. The inverse association between VD₃ and AD remained consistent across all subgroups, with no evidence of significant interaction (p for interaction > 0.05), indicating a robust and broadly applicable relationship.

## Discussion

Vitamin D is not only essential for skeletal health but also plays an important role in other crucial physiological functions. 1,25-dihydroxyvitamin D could alleviate chronic inflammatory states,and regulate the renin–angiotensin system, helping to lower blood pressure and reduce the risk of cardiovascular disease (19). These mechanisms collectively suggest that vitamin D may have a protective role in preventing inflammation-related diseases such as Alzheimer’s disease.

In this large, population-based study of Chinese female centenarians, we found a robust inverse association between serum 25-hydroxyvitamin D [25(OH)D] levels and the odds of Alzheimer’s disease (AD). This association remained statistically significant after adjusting for a wide range of demographic, clinical, and biochemical covariates, indicating that vitamin D may serve as an independent protective factor against AD in the oldest-old population. Notably, our analysis revealed a clear dose–response relationship, with progressively lower odds of AD observed across increasing quartiles of serum 25(OH)D. Using restricted cubic spline modeling, we further identified a potential threshold effect, whereby the protective association was most pronounced at serum 25(OH)D levels below approximately 29.3 ng/mL and plateaued thereafter. Subgroup analyses stratified by smoking, hypertension, and early-life socioeconomic and nutritional indicators (B8A–B10B) demonstrated consistent associations across strata, with no significant interactions, supporting the robustness and generalizability of the observed relationship.

Consistent with European and US cohorts (20) we observed an inverse association between serum 25(OH)D and AD risk, supporting VD₃’s role in attenuating neurodegeneration. A meta-analysis of 12 prospective studies (*N* = 65,000) similarly concluded that each 10 ng/mL increase in 25(OH)D reduces AD risk by 20% (HR = 0.80, 95% CI: 0.73–0.88) (21), aligning with our linear dose–response trend below the 30 ng/mL threshold. Mechanistically, VD₃’s anti-inflammatory effects—demonstrated by reduced plasma IL-6 (−18%, *p* = 0.03) in our high VD₃ subgroup—resonate with experimental models showing VD₃ suppresses microglial activation and Aβ-induced neurotoxicity (22–24). However, our study reveals unique features of VD₃ biology in centenarians. First, the nonlinear threshold effect contrasts with the linear associations reported in younger cohorts (e.g., Framingham: HR = 0.89 per 5 ng/mL) (8). This divergence may reflect age-related shifts in VD₃ metabolism: centenarians exhibit 40% lower dermal 7-dehydrocholesterol levels than octogenarians (25), potentially elevating the VD₃ threshold for neuroprotection. Second, unlike Japanese longevity cohorts where marine-derived VD₃ intake dominates (26), our Chinese cohort relied on solar synthesis (85% of 25(OH)D variance explained by UV exposure), suggesting population-specific pathways to VD₃ sufficiency.

Vitamin D₃ (VD₃) exerts neuroprotective effects through interconnected pathways that remain operative even in extreme longevity, with emerging evidence highlighting its role in Alzheimer’s disease (AD) pathogenesis via immune regulation, calcium homeostasis, and *β*-amyloid (Aβ) clearance (26, 27). First, VD₃ modulates immune responses linked to AD risk, as supported by studies demonstrating that immunoglobulin-related biomarkers (e.g., *κ*-chain fragments) enhance Aβ phagocytosis (28). Britschgi et al. (29) showed that immunoglobulin light chains promote Aβ clearance in murine models, a pathway potentially augmented by VD₃ in centenarians. Second, VD₃ regulates neuronal calcium signaling through L-type voltage-gated calcium channels (LTCCs) and transient receptor potential vanilloid 6 (TRPV6), as demonstrated by Brewer et al. (30). Schneider et al. (31) further reported a U-shaped relationship between serum calcium levels and dementia risk in older adults, where both hypocalcemia and hypercalcemia increased AD risk. Magnesium (Mg^2+^) levels were protective, consistent with VD₃’s role in balancing Ca^2+^/Mg^2+^ ratios critical for synaptic plasticity (32). Third, bone-derived hormones like osteocalcin (OST) mediate VD₃’s cognitive effects. Khrimian et al. (33) demonstrated that OST crosses the blood–brain barrier to enhance hippocampal BDNF expression in mice. Xiao et al. (34) identified a significant association between reduced bone mineral density and elevated dementia risk in a large prospective cohort, highlighting the role of skeletal health in neurodegenerative vulnerability. Fourth, the interplay between parathyroid hormone (PTH) and vitamin D₃ is critically implicated in Alzheimer’s disease pathogenesis. Elevated PTH levels disrupt neuronal calcium homeostasis, promoting amyloid-*β* aggregation, as demonstrated in both clinical cohorts and experimental models (35). Emerging evidence suggests that elevated serum phosphate levels, even within the physiological range, may contribute to neurodegenerative pathology through vascular calcification pathways, while longitudinal studies reveal that exceptional longevity is associated with lower phosphate homeostasis, possibly attenuating age-related vitamin D3 dysregulation (36). Multiple studies have identified early-life body size and socioeconomic status (SES) as significant determinants of dementia risk, acting through intertwined biological and social mechanisms (37, 38). For instance, research indicates that childhood SES is associated with cognitive impairment in older adulthood, with personality traits like conscientiousness and neuroticism mediating this relationship. Additionally, early-life factors such as education level, leg length, and childhood health conditions have been linked to the risk of dementia and cognitive impairment in later life (39). These findings underscore the importance of early-life conditions in influencing cognitive health outcomes (37).

The threshold of 29.3 ng/mL for serum 25-hydroxyvitamin D [25(OH)D] was identified through restricted cubic spline (RCS) modeling, which allowed us to explore potential nonlinear associations between vitamin D levels and Alzheimer’s disease (AD) risk. The inflection point was determined using likelihood-based methods to detect a change in slope in the dose–response relationship, suggesting a threshold effect. Below this cutoff, higher vitamin D levels were significantly associated with reduced odds of AD; however, this protective association plateaued beyond the threshold, indicating a diminishing return at higher concentrations.

Our finding is supported by previous literature suggesting that 25(OH)D levels ≥30 ng/mL may be necessary to achieve optimal extraskeletal effects, including neuroprotection and anti-inflammatory actions (21). In particular, Giustina et al. (25) emphasize that for older adults, higher vitamin D levels may be required to compensate for reduced dermal synthesis and altered metabolic clearance. Moreover, a recent cohort study by Geng et al. (20) observed a similar non-linear association between 25(OH)D and dementia, with attenuation of benefit above 30 ng/mL, reinforcing our threshold determination.

Our findings underscore the potential clinical relevance of maintaining adequate serum vitamin D₃ levels to mitigate Alzheimer’s disease (AD) risk, even in the context of extreme aging. Given the observed inverse and dose-responsive association between 25(OH)D and AD, routine screening and correction of vitamin D deficiency may represent a feasible and low-cost strategy to support cognitive health in the oldest-old. These results also highlight the value of incorporating vitamin D and related biomarkers—such as calcium, magnesium, PTH, and osteocalcin—into comprehensive cognitive surveillance programs. Future prospective cohort studies and randomized controlled trials are warranted to confirm the causal role of vitamin D₃ in AD prevention and to determine optimal therapeutic thresholds. Special emphasis should be placed on centenarians and high-risk elderly populations, for whom data remain scarce yet clinically informative.

This study has several notable strengths. First, it utilizes data from a well-characterized and rare cohort of Chinese female centenarians, allowing for unique insights into modifiable risk factors for Alzheimer’s disease (AD) in extreme longevity. Second, the analysis comprehensively adjusted for a wide range of potential confounders, including demographic factors, early-life exposures, and biochemical markers, thereby enhancing internal validity. Third, we employed advanced statistical techniques, including restricted cubic spline (RCS) models and piecewise logistic regression, to capture potential non-linear and threshold effects of serum vitamin D₃ [25(OH)D] on AD risk. Finally, subgroup analyses were conducted across multiple strata (e.g., smoking, hypertension, early-life socioeconomic indicators) to assess the robustness and consistency of the observed associations. However, several limitations should be acknowledged. The cross-sectional design precludes any inference of causality between vitamin D₃ levels and AD, as reverse causation cannot be excluded. Additionally, certain exposures (e.g., smoking history, early-life conditions) were self-reported, introducing the potential for recall bias. Finally, the study population consisted exclusively of female centenarians, which limits the generalizability of the findings to males or to younger elderly populations.

Our findings highlight the importance of adequate vitamin D status not only as a neurological factor, but also as a critical nutritional determinant of healthy aging. As vitamin D deficiency is highly prevalent among the oldest-old, our study underscores the need for age-tailored nutritional strategies. Ensuring sufficient vitamin D intake may represent a cost-effective public health approach to preserving cognitive function. These results support broader efforts in geriatric nutrition policy and reinforce the role of vitamin D as an essential component of comprehensive aging-related nutritional guidelines.

## Conclusion

This study provides novel evidence that higher serum vitamin D₃ levels are inversely associated with Alzheimer’s disease (AD) risk in female centenarians, independent of demographic, clinical, and biochemical factors. These results underscore the need to consider vitamin D₃ not only as a nutritional factor, but also as a potential regulator of multisystem resilience in the context of cognitive aging. Prospective studies are needed to confirm these associations and to explore whether optimizing vitamin D₃ status can contribute to dementia prevention in the oldest-old.

## Data Availability

The original contributions presented in the study are included in the article/supplementary material, further inquiries can be directed to the corresponding authors.
